# Adverse cardio-metabolic risk profile and epicardial adiposity in patients with adrenal adenoma: a cross-sectional study

**DOI:** 10.3389/fendo.2026.1898271

**Published:** 2026-08-05

**Authors:** Helena Niziolek, Ivica Just, Anna Tosin, Clemens Baumgartner, Oliver Koldyka, Konrad Körmöczi, Paul Fellinger, Hannes Beiglböck, Luise Bellach, Oliver Domenig, Maximilian Zeyda, Hansjörg Habisch, Tobias Madl, Anton Luger, Greisa Vila, Siegfried Trattnig, Alexandra Kautzky-Willer, Thomas Scherer, Michael Leutner, Michael Krebs, Martin Krssak, Peter Wolf

**Affiliations:** 1 Division of Endocrinology and Metabolism, Department of Internal Medicine III, Medical University of Vienna, Vienna, Austria; 2 Attoquant Diagnostics, Vienna, Austria; 3 Division of Pediatric Pulmonology, Allergology and Endocrinology, Department of Pediatrics and Adolescent Medicine, Medical University of Vienna, Vienna, Austria; 4 Division of Medicinal Chemistry, Otto Loewi Research Center for Vascular Biology, Immunology and Inflammation, Medical University of Graz, Graz, Austria; 5 BioTechMed-Graz, Graz, Austria; 6 Department of Biomedical Imaging and Image Guided Therapy, Medical University of Vienna, Vienna, Austria

**Keywords:** adrenal adenoma, cardiometabolic risk, cross-sectional analysis, hepatic lipid accumulation, mild autonomous cortisol secretion

## Abstract

**Background:**

Mild autonomous cortisol secretion (MACS) is associated with an increased cardiovascular mortality, but even patients with non-functioning adrenal adenoma (NFA) might be at risk for cardio-metabolic comorbidities compared to a control group with absence of an adrenal tumor (CON).

**Material and methods:**

Seventyfour individuals were included in cross-sectional analysis [MACS: n=25(84% females), age: 63(55-67)years, BMI:27.00(24.20-30.50)kg/m^2^; NFA: n=25(84%females), age: 61(54-68)years, BMI:28.50(27.50-32.20)kg/m^2^ and CON: n=24 (75%females), age: 58 (51-64)years, BMI: 27.39(22.03-30.17) kg/m^2^]. Visceral and ectopic fat stores, as well as heart function were assessed by magnetic resonance imaging and spectroscopy. Blood pressure was measured by standardized protocols. Blood was drawn in the fasting state and during an oral glucose tolerance test to assess glucose and lipid metabolism.

**Results:**

The prevalence of hypertension (MACS:84% *vs* NFA:76% *vs* CON: 43.5%), dyslipidemia (MACS:96% *vs* NFA:80% *vs* CON:62.5%) and prediabetes (MACS:56% *vs*. NFA:72% *vs* CON:29.2%) was significantly different between the groups. Markers of glucose metabolism were worse in MACS and best in CON (HbA1c: 5.70 (5.40-5.90)% *vs* 5.50 (5.40-5.80)% *vs* 5.40 (5.27-5.60)%, p=0.043); HOMA_IR:2.91(2.19-4.15) *vs* 2.55 (1.57- 4.97) *vs* 1.73 (1.00-2.61), p=0.007). epicardial adipose tissue area was highest in MACS patients, followed by NFA, with lowest values in CON (MACS:1159.68 (930.38-1454.11) mm^2^ *vs* NFA: 873.23 (638.03-1195.33) mm^2^ *vs* CON:672.37 (549.44-890.30) mm^2^, p<0.001). No relevant between-group-differences in cardiac morphology, intramyocardial fat or intrahepatic fat were observed.

**Conclusions:**

In this exploratory study, MACS was associated with epicardial adiposity and an adverse cardio-metabolic risk profile.

## Introduction

Adrenal incidentaloma are common in the general population with an estimated prevalence of 1.4 – 7%, increasing with age. While most of these tumors can be classified as hormonally inactive non-functioning adenomas (NFA) without the need for further follow up, 19-50% of patients will be diagnosed with mild autonomous cortisol secretion (MACS). MACS therefore represents the most common hormone abnormality in these patients (1).

MACS is defined by a morning serum cortisol concentration >1.8 µg/dL after the 1 mg dexamethasone suppression test (DST) in the absence of classical clinical features of Cushing’s syndrome (2). Underestimated as “subclinical” disease in the past, there is nowadays robust evidence showing that MACS is associated with an increased morbidity and mortality, mainly because of cardio-metabolic diseases (3). Interestingly, even patients with NFA might exhibit an increased risk for metabolic comorbidities (4), as well as an increased cardiovascular mortality compared to healthy individuals with absence of adrenal tumors (5). However, the prevalence of cardio-metabolic adverse events is much higher in MACS compared to NFA (6, 7), with a linear association between mortality and cortisol values after DST (8).

Epicardial adipose tissue might be an important driver for the increased cardiovascular risk in patients with MACS. Given its proximity to the myocardium and the coronary arteries, epicardial fat promotes myocardial fibrosis, endothelial and contractile dysfunction and atherosclerosis by the paracrine release of pro-inflammatory cytokines (9–11). While epicardial fat is strongly associated with the presence of hypercortisolism in patients with ACTH dependent Cushing’s syndrome (12, 13), data regarding an accumulation of epicardial adipose tissue in patients with adrenal incidentalomas is conflicting (14–16). This discrepancy is probably best explained by technical limitations as epicardial adipose tissue has up to now only been assessed by echocardiography in patients with MACS. Cardiac and hepatic steatosis might also be of relevance, given their close association with type 2 diabetes mellitus, dyslipidemia and visceral obesity in the general population (17, 18).

This study therefore aimed to perform an in-depth cardio-metabolic characterization of patients with MACS and to compare them to patients with NFA and controls without a known adrenal adenoma (CON), using magnetic resonance (MR) imaging and spectroscopy techniques combined with blood tests.

## Materials and methods

This is an exploratory, cross-sectional cohort study, conducted as single site study at the Division of Endocrinology and Metabolism at the Medical University of Vienna. The study was approved by the Ethics committee of the Medical University of Vienna (1062/2022) and registered at EudraCT (2022-000161-40). The control group without a known adrenal adenoma was pooled from previously performed studies (19), which were all approved by the Ethics committee of the Medical University of Vienna (IntoHumans 2024/2013; RISC and CaSR 1128/2013; Fructose 1022/2013; EpiTyp-2: 2197/2015, HYPO 1288/2012).

### Participants

A total of 74 participants were enrolled in the study, 25 patients with adrenal adenoma and MACS (MACS; 21 females, 4 males), 25 patients with non-functioning adrenal adenoma (NFA; 21females, 4 males) and 24 control subjects without a known adrenal adenoma (CON; 18 females, 6 males).

Adult patients with uni- or bilateral adrenal adenoma(s) confirmed by computed tomography or magnetic resonance imaging prior to study entry were included. All patients with adrenal adenoma underwent routine hormonal work-up including a 1mg dexamethasone suppression test (DST). MACS was diagnosed in patients with cortisol values >1.8 µg/dL without clinical signs of Cushing’s syndrome according to recent guidelines. Patients with adequate suppression of cortisol after DST were considered to have a non-functioning adrenal adenoma (NFA) (2). Exclusion criteria for adrenal adenoma patients comprised daily intake of more than four antihypertensive agents, requirement of insulin therapy, poorly controlled diabetes with a HbA1c >8%, kidney disease (eGFR CKD-EPI<45 mL/min/1.73m^2^) or liver disease (AST/ALT elevations >3× ULN), malignancy features of adrenal masses, pregnancy, breastfeeding, regular administration of potent *CYP3A4* modulators or glucocorticoids within three months prior to study inclusion.

Exclusion criteria for the pooled controls were history of an adrenal adenoma intake of medication affecting glucose or lipid metabolism; history of cardiovascular, liver or renal disorders; any acute inflammatory disease within 2 weeks prior to the study; pregnancy and nursing; and any MR contraindications including claustrophobia, metal devices in or on the subject’s body. Controls were selected retrospectively from an available pool of previously investigated participants by a member of the study team (IJ) not involved in clinical evaluation or data analysis. Results of MR measurements and clinical data were not reviewed prior to the matching procedure in order to minimize selection bias. The total data set consisted of 503 individuals (19). Potential CON participants were first screened for availability of both abdominal and cardiac MRI datasets, which were available for 409 individuals. After applying exclusion criteria, eligible participants were matched on a one-to-one basis to MACS patients first by age, followed by BMI and subsequently by sex. As most individuals of the retrospective control cohort were substantially younger than the patients in the MACS and NFA cohorts, only 24 control subjects without a history of adrenal adenoma were eligible for further analysis. Of note, no CT or MRI scan of the adrenal glands was routinely performed in the control cohort. Furthermore, control subjects had no results of a 1mg DST or baseline ACTH measurements available.

### Procedures and outcomes

Patient history and regular medication were obtained at study inclusion with expanded information retrieved from patient charts. Anthropometric parameters (height, weight, waist circumference) were measured at the study day.

Blood pressure was measured after at least 5 minutes of rest in supine position. Hypertension was defined by regular intake of antihypertensive agents or blood pressure values ≥140/90 throughout the study procedures.

Blood was drawn after an overnight fast to examine the following parameters by routine laboratory methods using immunoassays applying standardized and validated measurement procedures(https://labormedizin.meduniwien.ac.at/): electrolytes, hormone parameters of the hypothalamic-pituitary-adrenal (HPA) axis, parameters of glucose and lipid metabolism, kidney- and liver function parameters.

An oral glucose tolerance test (OGTT) was performed with consecutive blood samples for assessment of glucose, insulin and c-peptide every 30 minutes for 2 hours after ingestion of 75 g glucose. OGIS Calculator:0-90-120min(http://webmet.pd.cnr.it/ogis/ogis.php) was used for outcome calculation of OGIS120. HOMA_IR was calculated using the formula:(fasting glucose x fasting insulin)/405.

Prediabetes was defined as a HbA1c value of 5.7-6.4% or impaired glucose tolerance during OGTT with fasting glucose values between 100-125mg/dL or 2h OGTT glucose values between 140-199mg/dL. Diabetes mellitus was determined by regular intake of antidiabetic medication, a HbA1c ≥ 6.5% or in case of fasting glucose ≥ 126 mg/dL or 2h-OGTT values ≥200mg/dL.

Dyslipidemia was identified by regular intake of lipid-lowering agents or abnormal laboratory findings of at least one lipid parameter (total cholesterol >200 mg/dL, triglycerides >150 mg/dL in the fasting state, LDL cholesterol >130 mg/dL, HDL cholesterol <40 mg/dL in male participants and <50 mg/dL in female participants) (20).

### Branched-chain amino acids

A Shimadzu 8050 LCMS using MassChrom^®^ Amino Acid Kit (Chromsystems, Munich, Germany) was used to obtain concentrations of valine, leucine, isoleucine and L-alloisoleucine (21). We report both individual values and the calculated sum of total obtained analyte concentrations from valine, isoleucine and leucine.

### Body composition

In patients with an adrenal incidentaloma (MACS and NFA) body composition was examined with a BODPOD system (BOD POD GS-X, Cosmed, Rome, Italy), an air displacement plethysmography device (22).

### Renin-angiotensin-aldosterone system

Angiotensin I, angiotensin II and aldosterone equilibrium concentrations were measured after equilibration and stabilization of conditioned samples (23, 24). Samples were supplemented with stable isotope-labeled internal standards for individual angiotensins (200 pg) and with deuterated (D4) aldosterone (500 pg). Liquid chromatography tandem mass spectrometry was performed with a reversed-phase analytical column(Acquity UPLC^®^ C18, Waters) complying with a XEVO TQ-S triple quadrupole mass spectrometer (Waters Xevo TQ/S, Milford, Massachusetts, USA) using multiple reaction monitoring. Standardized correction was performed in each sample for analyte recovery during sample handling. Individual concentrations were obtained from integrated chromatograms taking into account corresponding response factors based on calibration curves in serum matrix, if the integrated signals exceeded a signal-to-noise ratio of 10. For determination of RAAS triple A activity equilibrium concentrations of angiotensin I, angiotensin II and aldosterone levels were obtained as well as markers for plasma-renin-activity (PRA-S: angiotensin I + angiotensin II), angiotensin-converting-enzyme (ACE-S: angiotensin II/angiotensin I) and the AA2-Ratio (aldosterone/angiotensin II), a marker for adrenal responsiveness to angiotensin II mediated aldosterone release, was estimated.

### Lipidomics

Bruker 600 MHz Avance Neo NMR spectrometer (Bruker, Rheinstetten, Germany) served for analysis of serum samples. Samples were thawed, 300µl incorporated with 300µl Bruker buffer and processed following the Bruker *In Vitro* Diagnostics research workflow with subsequent spectra analysis in TopsPin 4.4 and quantified with the Bruker Online Server using B.I.LISA for lipoprotein subclass analysis.

### Magnetic resonance imaging and spectroscopy techniques

^1^H-magnetic resonance spectroscopy (^1^H-MRS) and imaging measurements of all included patients were conducted with a high-field 3 Tesla whole body MR system (Magnetom, Prisma Fit, Siemens Healthineers, Erlangen, Germany) including a spine and body coil from Siemens Healthineers, Erlangen Germany. As published before, intrahepatic lipid content (IHL) was measured with single voxel ^1^H-MRS (based on methylene and methyl resonance signals corrected for longitudinal and individual transversal relaxation times) and determined as percentage of total MRS signal encompassing water, methylene and methyl peaks (25).

Cardiac measurements were performed after full exhalation with breath hold: ECG-gated cine true fast imaging with steady-state precession (TrueFISP) protocols were carried out to evaluate cardiac function. Images were obtained in standard, two-chamber, four-chamber, and short-axis orientations, with 10–12 slices extending from the apex to the base of the left ventricle. Analysis of ejection fraction, peak ejection rates, cardiac output, stroke volume, left ventricular myocardial mass, end-systolic volume and end-diastolic volume, and filling rates was performed. To obtain cardiac function outcomes, short axis images over the total cardiac phases were analyzed via syngo.via software (Siemens Healthineers, Erlangen, Germany) through semi-automated delineation of endocardial and epicardial borders.

For estimation of intramyocardial fat deposition, a volume of interest was placed at the interventricular septum: Within a single breath hold, a PRESS sequence was performed for signal acquisition and positioning. Spectroscopy software by the manufacturer Siemens Healthineers was applied for spectral data analysis. Intramyocardial lipid content was quantified by the ratio of combined signal intensities of methylene and methyl resonance to the water signal and reported as percentage to total signal (fat fraction). T_1_ and T_2_ relaxation times corrections were applied with reference values of the skeletal muscle (26).

### Epicardial and paracardial adipose tissue

Visual determination of long-axis four-chamber MRI slice was performed in three time points of the cardiac cycle phase: The end-diastolic phase marks the largest ventricular expansion, the end-systolic phase the smallest ventricular expansion- the third slice marks the intermediate phase. Paracardial fat is attached outside of the pericardium, while epicardial fat is situated between the myocardium and the pericardium. We outlined the borders of fat deposition visually in described cardiac phases and mapped them with FIJI software (27, 28). All three epicardial and paracardial areas were summed up and for each fat type the average was calculated per patient.

Analysis of MR imaging and spectroscopy measurements was done by study team members blinded to the clinical and metabolic results (IJ, MKRS). Epicardial/paracardial fat areas were assessed by an unblinded analyst (HN), but both inter and intra-observer reproducibility were crosschecked at initial steps of analysis.

### Statistical analysis

This is an exploratory analysis of prespecified outcome parameters. No formal sample size calculation was performed for the included parameters given the scarcity of available literature for ectopic fat in patients with MACS. The target sample size of 25 patients per group was based on previous study protocols (25–27) investigating comparable outcomes. Intrahepatic lipid content was defined as the primary outcome parameter. All other outcome parameters were prespecified as secondary outcome parameters in the study protocol (Eudra-CT 2022-000161-40). Quantitative data was reported with medians (Q1-Q3), while qualitative data was presented with absolute numbers (percentages). For correlation analysis, following a non-parametric approach, Spearman’s correlation coefficient was applied. For intergroup comparison both Mann Whitney-U tests and Kruskal Wallis tests were used depending on available cohort number. Categorical variables were compared by Chi-square tests or Fisher’s exact tests were appropriate. *Post hoc* Dunn’s tests were performed for variables with significant group differences in the Kruskal Wallis test. Both unadjusted and Holm-adjusted p-values were calculated and reported. Linear regression models were fitted with clinically relevant variables included as covariates. Sensitivity analysis of regression models was performed using heteroscedasticity robust standard errors. To meet regression model assumptions, right skewed parameters were log2-transformed. In addition, variance inflation factors were calculated to explore multicollinearity. Differences were considered significant at p-values <0.05. Statistical analysis was performed using R version 4.4.1 (2024-06-14).

## Results

### Study population

Seventy-four subjects were included in cross-sectional analysis: 25 patients with MACS, 25 patients with NFA and 24 CON. Participants were of comparable age, sex and BMI across the three groups according to our inclusion criteria (**Table 1**).

**Table 1:** Data are reported as medians (q1-q3) for continuous variables and total numbers (percentages) for dichotomous variables.

Clinical characteristics	Number of available values per group MACS/NFA/CON	MACS	Non-functioning adenomas	Controls	p-value
n		25	25	24	
Age	25/25/24	63 (55, 67)	61 (54, 68)	58 (51, 64)	0.124
Sex (female)	25/25/24	21(84%)	21(84%)	18(75%)	0.655
BMI kg/m2	25/25/24	27.00 (24.20, 30.50)	28.50 (27.50, 32.20)	27.39 (22.03, 30.17)	0.273
Weight, kg	25/25/23	71.60 (64.70, 81.97)	75.65 (68.53, 85.99)	79.80 (59.30, 89.10)	0.732
Systolic blood pressure, mmHg	25/25/22	128.00 (121.00, 141.00)	133.00 (125.00, 144.00)	137.50 (116.66, 141.00)	0.756
Diastolic blood pressure, mmHg	25/25/22	83.00 (78.00, 94.00)	83.00 (77.00, 92.00)	80.00 (70.33, 88.41)	0.247
Heart rate, bpm	24/25/0	63.50 (56.75, 70.25)	63.00 (61.00, 68.50)	NA	0.581
Unilateral adenomas	25/25/0	13(52%)	24(96%)	NA	0.001
Diameter in mm	25/25/0	23.00 (17.50, 30.00)	16.00 (11.00, 20.00)	NA	0.005
Cortisol after 1mg DST, µg/dL	25/25/0	3.10 (2.40, 5.09)	1.10 (0.80, 1.30)	NA	<0.001
Morning serum-Cortisol, µg/dL	24/25/9	13.00 (10.20, 16.05)	14.40 (12.90, 15.70)	12.30 (10.30, 16.00)	0.802
ACTH, pg/mL	25/25/0	4.00 (4.00, 7.00)	19.00 (15.00, 28.00)	NA	<0.001
DHEAS, µg/mL	24/25/7	0.60 (0.31, 0.78)	0.90 (0.52, 1.21)	1.86 (1.23, 3.29)	0.001
Prevalence of hypertension	25/25/23	21(84%)	19(76%)	10(43.5%)	0.006
Number of antihypertensive agents	25/25/0	2.00 (1.00, 3.00)	1.00 (0.00, 2.00)	NA	0.090
Prevalence of prediabetes	25/25/24	14(56%)	18(72%)	7(29.2%)	0.010
Prevalence of diabetes mellitus type 2	25/25/24	5(20%)	2(8%)	0(0%)	0.062
Prevalence of dyslipidemia	25/25/24	24 (96%)	20(80%)	15(62.5%)	0.014

Intergroup differences were calculated applying Kruskal Wallis test (continuous variables) or chi square test/fisher exact test depending on expected cell count(dichotomous variables); Mann Whitney U tests were applied for pairwise comparison of patients with MACS and non-functioning adenomas. BMI, body mass index; DST, dexamethasone suppression test; ACTH, adrenocorticotropic hormone, DHEAS, dehydroepiandrosterone sulfate.

MACS patients were more likely to present with bilateral adenomas and a larger diameter of the adrenal mass compared to NFA patients. Serum cortisol concentrations after 1 mg DST were higher and ACTH levels were significantly lower in MACS compared to NFA. Comparison of all three groups displayed significant intergroup differences for DHEAS, with lowest median values in the MACS cohort and highest in the control group. Dunn *post hoc* procedure displayed significantly lower DHEAS levels in MACS compared to NFA and CON(both unadjusted and Holm adjusted p-values). No significant alterations of DHEAS levels were determined between NFA and CON (**Supplementary Table 1**).

Systolic and diastolic blood pressure were not differing between the groups, but the prevalence rates of hypertension and dyslipidemia declined numerically from MACS to NFA and were lowest in CON (**Table 1**; **Supplementary Table 2**). Clinical characteristics and parameters on hypothalamus-pituitary-adrenal axis activity are displayed in detail in **Table 1**.

### Parameters of cardiac fat depots, cardiac function & - morphology

Data on cardiac fat depots, cardiac function & - morphology are reported in detail in **Table 2** and **Supplementary Table 1**.

**Table 2:** Comparison of was performed variables using Kruskal Wallis test (MACS vs NFA vs CON).

Cardiac MRI outcome variables	Number of available values per group MACS/NFA/CON	MACS (Median, q1-q3)	Non-functioning adenoma (Median, q1-q3)	Controls (Median, q1-q3)	p-value
MRI measurements	n=				
Intramyocardial lipid content, %	22/25/24	0.54 (0.30, 0.81)	0.50 (0.35, 0.81)	0.42 (0.28, 0.73)	0.700
Mean epicardial adipose tissue, mm2	24/25/19	1159.68 (930.38, 1454.11)	873.23 (638.03, 1195.33)	672.37 (549.44, 890.30)	<0.001
Mean paracardial adipose tissue, mm2	24/25/19	1242.62 (967.20, 1606.67)	1118.83 (856.27, 1562.00)	713.70 (540.88, 1249.82)	0.032
EF, %	24/25/19	61.52 (56.05, 64.98)	60.80 (57.89, 65.59)	61.90 (58.03, 64.58)	0.994
EDV, ml/m2	24/25/18	64.03 (59.17, 78.14)	67.15 (59.59, 77.02)	57.48 (47.55, 70.75)	0.092
ESV, ml/m2	24/25/18	26.37 (22.94, 29.43)	26.42 (22.75, 32.37)	20.79 (18.85, 31.38)	0.409
Stroke, ml/m2	24/25/18	39.34 (34.64, 45.82)	40.95 (36.68, 47.15)	34.36 (28.70, 41.76)	0.026
Cardiac index, L/min/m2	24/25/18	2.54 (2.25, 2.93)	2.66 (2.54, 3.02)	2.37 (2.01, 2.74)	0.103
Left ventricle myocardial mass average g/m2	24/25/17	61.59 (52.64, 67.19)	62.23 (57.87, 63.99)	59.70 (56.31, 67.50)	0.989

P-values were rounded to three decimals. Intramyocardial lipid fraction (%) was calculated with the formula: (lipid peak area/(lipid peak area +water peak area))x100. Cardiac morphology and function parameters were normalized to body surface area (BSA). Data on epicardial and paracardial adipose tissue was available in 19 controls. EF, ejection fraction; EDV, end-diastolic volume; ESV, end-systolic volume.

**Figure 1:**
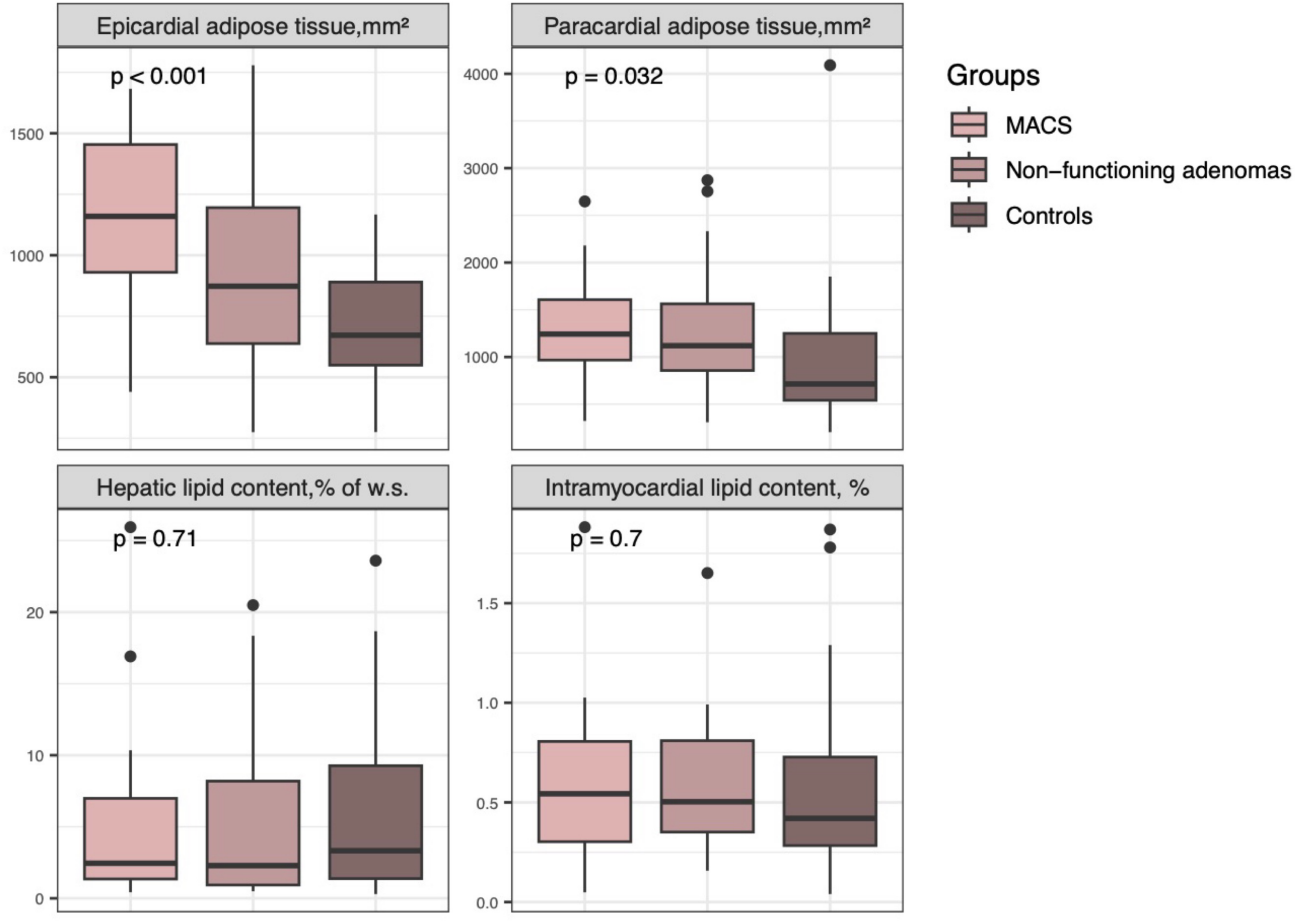
Comparisons of ectopic fat depots among the three cohorts(MACS vs NFA vs CON) were performed using the Kruskal Wallis test. Significant variation of epicardial adipose tissue(EAT, p<0.001) and paracardial adipose tissue (PAT, p=0.032) was determined, while no alterations of hepatic lipid content and intramyocardial lipid content were found. The number of available observations (MACS/NFA/CON) was as follows: epicardial and paracardial fat, 24/25/19; hepatic lipid content, 25/25/24; and intramyocardial lipid content, 22/25/24. (w.s.,water signal).

In 5 CON subjects, epicardial and paracardial adipose tissue areas could not be assessed because of reduced imaging quality or availability. Significant differences in epicardial adipose tissue area were identified, with decreasing fat accumulation from MACS to NFA and then further to CON. In unadjusted Dunn’s *post hoc* tests assessing epicardial fat area, significant differences were found in pairwise comparisons among all three groups. However, after Holm-adjustment, epicardial adipose tissue was higher in MACS vs CON and NFA vs CON, while comparison of MACS vs NFA was not significant (**Figure 1**; **Supplementary Table 1**).

Paracardial adipose tissue area was higher in MACS compared to CON in both unadjusted and adjusted analyses, while the remaining pairwise comparisons were not significant (**Supplementary Table 1**).

Lowest median value of stroke volume was outlined in CON, but otherwise no inter-group disparities in cardiac function and morphology were observed.

### Parameters of glucose and lipid metabolism, branched chain amino acids and RAAS activity

**Figure 2:**
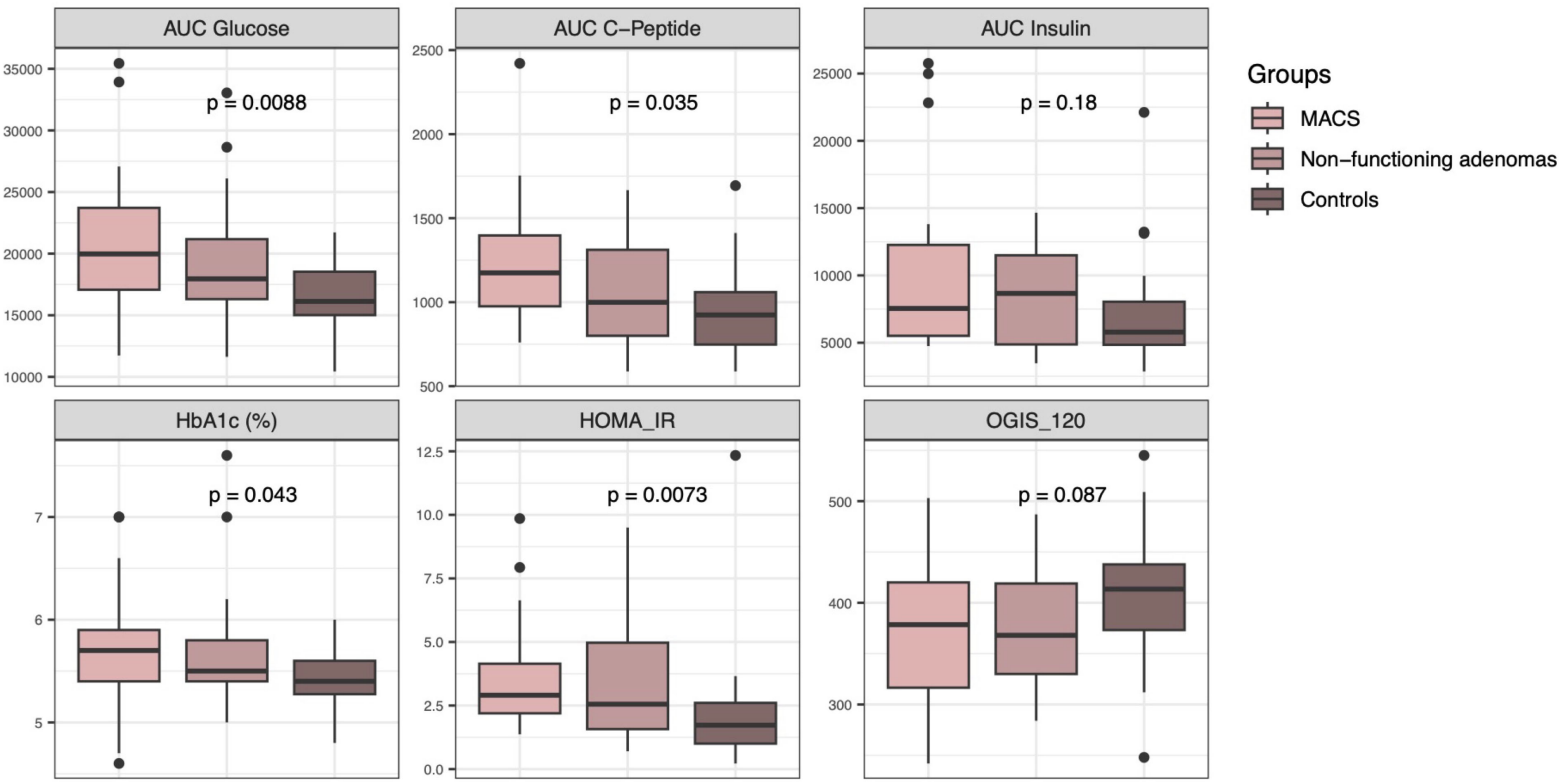
Calculation of intergroup differences among MACS,NFA and CON in markers of glucose homeostasis using the Kruskal Wallis test: A significant alteration in AUC of glucose (p=0.0088), AUC of c-peptide (p=0.035), HbA1c (p=0.043) and HOMA_IR (p=0.0073) was observed. No disparities in AUC of insulin (p=0.18) and OGIS_120 were detected (p=0.087). The available value number per group (MACS/NFA/CON) is as follows: HbA1c, 25/25/24; HOMA-IR, 25/24/24; AUC glucose, 22/23/23; AUC insulin, 22/23/23; AUC C-peptide, 22/23/22; and OGIS, 24/23/20. AUC, area under the curve; HOMA_IR, HOMA_IR, Homeostasis model assessment of insulin resistance;OGIS_120, Oral Glucose Insulin Sensitivity.

Data on glucose and lipid metabolism are reported in detail in **Table 3**; **Figure 2**.

**Table 3:** All outcome parameters are listed with medians (q1-q3); p-values were calculated using the Kruskal Wallis test (MACS vs. NFA vs CON).

Metabolic and hormone outcome parameters	Number of available values per group MACS/NFA/CON	MACS	Non-functioning adenoma	Controls	p-value
Waist Circumference, cm	24/25/21	97.50 (91.88, 104.00)	100.00 (93.00, 108.00)	96.00 (80.00, 103.00)	0.345
Body fat, %	25/25/0	45.10 (34.90, 49.70)	41.10 (36.20, 48.00)	NA	0.691
Total body fat mass, kg	25/25/0	31.20 (21.39, 39.26)	33.10 (24.45, 39.80)	NA	0.744
Total body fat free mass, kg	25/25/0	40.30 (37.82, 44.62)	42.24 (40.30, 51.17)	NA	0.224
Hepatic lipid content, % of w.s.	25/25/24	2.45 (1.35, 6.99)	2.28 (0.93, 8.19)	3.33 (1.38, 9.27)	0.708
HbA1c, %	25/25/24	5.70 (5.40, 5.90)	5.50 (5.40, 5.80)	5.40 (5.27, 5.60)	0.043
HOMA_IR	25/24/24	2.91 (2.19, 4.15)	2.55 (1.57, 4.97)	1.73 (1.00, 2.61)	0.007
Fasting insulin, µlU/mL	25/24/24	11.70 (9.07, 16.30)	10.55 (6.46, 19.50)	7.95 (4.68, 11.32)	0.014
Fasting C-peptide, ng/mL	25/24/24	2.90 (2.40, 4.20)	2.70 (1.90, 3.80)	2.15 (1.87, 2.82)	0.018
Fasting glucose, mg/dL	25/25/24	97.00 (93.00, 105.00)	95.00 (90.00, 98.00)	92.00 (86.75, 97.50)	0.051
OGIS,ml·min^−1^·m^−2^	24/23/20	378.50 (316.50, 420.00)	368.00 (330.00, 419.00)	413.50 (373.25, 437.75)	0.087
AUC Glucose, mg*min/dL	22/23/23	19980.00 (17073.75, 23715.00)	17955.00 (16312.50, 21172.50)	16125.00 (15015.00, 18532.50)	0.009
AUC Insulin, µlU*min/mL	22/23/23	7549.58 (5514.26, 12271.76)	8665.50 (4870.73, 11499.75)	5790.30 (4842.41, 8048.29)	0.176
AUC C-Peptide, ng*min/mL	22/23/22	1174.50 (975.38, 1397.25)	999.00 (800.25, 1311.75)	924.00 (747.75, 1059.75)	0.035
AST,U/L	22/21/24	21.00 (19.00, 26.25)	21.00 (19.00, 25.00)	22.50 (21.00, 25.25)	0.691
ALT, U/L	24/24/24	23.00 (19.00, 28.75)	21.50 (18.00, 30.75)	20.00 (17.75, 29.00)	0.389
GGT, U/L	24/24/24	27.00 (19.75, 41.75)	21.00 (14.00, 29.00)	17.50 (15.75, 21.50)	0.030
Total cholesterol, mg/dL	25/25/24	200.00 (167.00, 221.00)	189.00 (167.00, 219.00)	205.00 (175.25, 218.75)	0.635
HDL-cholesterol, mg/dL	25/25/24	61.00 (48.00, 73.00)	65.00 (50.00, 75.00)	51.50 (44.00, 68.50)	0.401
LDL-cholesterol, mg/dL	25/25/24	107.80 (70.40, 134.40)	95.00 (85.40, 133.60)	125.90 (93.05, 136.00)	0.350
Triglycerides, mg/dL	25/25/24	131.00 (90.00, 213.00)	97.00 (74.00, 146.00)	101.00 (75.25, 140.25)	0.028
Branched chain amino acids					
Allo-isoleucine, µmol/l	25/25/0	2.90 (2.40, 3.40)	2.80 (2.30, 3.10)	NA	0.567
Isoleucine, µmol/l	25/25/0	58.50 (51.10, 69.00)	58.20 (51.20, 69.70)	NA	0.938
Leucine, µmol/l	25/25/0	140.60 (119.10, 154.20)	142.60 (121.30, 153.70)	NA	0.908
Valine, µmol/l	25/25/0	284.90 (258.60, 325.90)	302.90 (254.65, 330.40)	NA	0.715
Sum: Isoleucin,Leucin and Valine, µmol/l	25/25/0	469.80 (436.17, 550.70)	504.50 (416.80, 549.20)	NA	0.818
Renin-angiotensin-aldosterone system					
Angiotensin II (1-8), pmol/L	25/25/0	53.90 (22.04, 89.19)	63.57 (22.24, 208.28)	NA	0.386
Angiotensin I (1-10), pmol/L	25/25/0	21.43 (13.40, 47.84)	42.87 (11.55, 141.04)	NA	0.189
Aldosterone, pmol/L	25/25/0	97.55 (76.56, 173.01)	186.45 (117.97, 251.45)	NA	0.041
Aldosterone-Angiotensin II-ratio	25/25/0	1.91 (0.95, 7.63)	2.60 (1.33, 9.15)	NA	0.863
PRA-S, pmol/L	25/25/0	81.10 (30.92, 142.37)	133.61 (47.45, 414.47)	NA	0.147
ACE-S	25/25/0	3.01 (1.98, 3.97)	2.79 (1.61, 4.16)	NA	0.985

Mann Whitney U test (MACS vs NFA) was applied if insufficient data amount (0–6 values per variable) were available for the control cohort. P-values were rounded to three decimals. ACE-S, angiotensin II/angiotensin I; AUC, area under the curve; HOMA_IR, homeostasis model assessment of insulin resistance; AST, aspartate aminotransferase; ALT, alanine aminotransferase; GGT, gamma-glutamyl transferase; HDL, high-density lipoprotein; LDL, low-density lipoprotein; PRA-S; angiotensin I + angiotensin II

Group comparison of HbA1c showed significantly higher HbA1c in MACS vs CON, while no differences were observed between MACS vs NFA and NFA vs CON in both unadjusted and Holm adjusted analysis. HOMA_IR was significantly higher in MACS compared to CON, while comparison of NFA vs CON was only significant in unadjusted analysis and no difference was outlined between MACS and NFA in adjusted and unadjusted Dunns’s tests. Fasting insulin was higher in MACS compared to CON, while only in unadjusted analysis NFA compared to CON was significantly higher. No difference was outlined between MACS and NFA in (un)-adjusted analysis.

Fasting C-peptide and paracardial fat area displayed higher values in MACS vs CON, while no differences were found in inter-group comparison of MACS vs NFA and NFA vs CON in both adjusted and unadjusted *post hoc* tests.

No differences in concentrations of total cholesterol, LDL-cholesterol and HDL-cholesterol were observed. Triglycerides were significantly higher in MACS vs CON/NFA in the unadjusted analysis. After Holm adjustment the difference remained only significant in MACS vs NFA, whereas no differences were observed in MACS vs CON and NFA vs CON. Lipidomics analysis was performed in patients with MACS and NFA displaying alterations in lipoprotein sub-components (**Supplementary Figure 1**).

Examination of angiotensin metabolites between MACS and NFA cohort displayed lower aldosterone concentration in the MACS cohort. No differences in concentrations of branched chain amino acids were found.

Body weight and waist circumference was comparable between the three groups. Body composition was available only for adrenal adenoma patients and was not different between MACS and NFA.

With regards to MR analysis, no difference in intrahepatic lipid content was observed between the three groups.

### Correlation analyses and regression models

Results of univariate correlation analyses of HOMA_IR and epicardial fat area with selected clinical and biochemical parameters are reported in the **Supplementary Table 3**.

In a second step, a linear regression model including group status(MACS vs NFA vs CON), age and waist circumference was fitted for epicardial adipose tissue. Compared to the reference group (MACS), epicardial fat was significantly lower in the NFA (β=-264.7, p=0.006) and CON (β= -344.3,p=0.002) cohort. Waist circumference was independently associated with epicardial adipose tissue area (β=11.6, p<0.001), while age was not significantly associated.

In a linear regression model including group allocation (MACS vs NFA vs CON), age and waist circumference, log(HOMA-IR) remained significantly lower in CON compared to the reference group (MACS) (β=-0.443, p = 0.014). No significant difference was observed between MACS and the NFA cohort (β=-0.223,p=0.178). Waist circumference was independently associated with log(HOMA_IR) (β=0.029,p<0.001), whereas no significant association was observed for age (**Supplementary Table 4**).

## Discussion

In this cross-sectional cohort study, we combined MR spectroscopy and imaging techniques with a comprehensive functional and biochemical cardio-metabolic assessment of patients with MACS, patients with NFA and CON without a known adrenal adenoma. We observed an increased accumulation of epicardial adipose tissue in MACS and NFA together with a worse metabolic profile.

The median amount of epicardial adipose tissue and paracardial adipose tissue area were highest in the MACS cohort and lowest in the control group. An accumulation of epicardial and paracardial fat mass was reported in patients with prediabetes/diabetes and obesity (29, 30). Furthermore, an increase in epicardial adipose tissue was observed in patients with Cushing’s syndrome (12, 13, 31), patients with adrenal adenoma and mild Cushing’s syndrome (15). We therefore assume that the presence of hypercortisolism, but also the adverse metabolic profile might both contribute to epicardial adiposity. The latter might also explain the observed increase in epicardial adipose tissue area in patients with NFA compared to CON.

Epicardial adipose tissue might be of particular relevance for the increased cardiovascular risk in patients with MACS as it is an independent risk factor for cardiovascular mortality (32). Besides promoting atherosclerosis (33), the paracrine secretion of pro-inflammatory adipocytokines from epicardial fat into the myocardium drives cardiac fibrosis, impairs myocardial contractility (11, 34) and results in endothelial dysfunction (35).

With regards to conventional cardiovascular risk factors, our cross-sectional analysis shows numerical differences, with the highest total prevalence of hypertension, diabetes mellitus type 2 and dyslipidemia in MACS and lowest in CON (**Table 1**; **Supplementary Table 2**). No differences in concentrations of branched-chain amino acids were observed between MACS and NFA.

The worse cardio-metabolic profile in MACS is in accordance with previous literature (3) and suggests a linear increase of comorbidities in adrenal adenoma patients together with increasing cortisol post DST values (8). Interestingly, comparison of patients with adrenal adenoma to a matched control cohort without a history of adrenal tumors showed a higher prevalence of hypertension, prediabetes and a worse glucose metabolism in NFA compared to CON. Prediabetes was even more prevalent in NFA compared to MACS. Overall, our data support the hypothesis of an increasing risk from CON to NFA to MACS, as suggested previously (4), and might indicate an adverse metabolic profile in NFA (5, 36). However, the overlap in metabolic outcomes between MACS and NFA patients might indicate shared metabolic disturbances across adrenal adenoma phenotypes. Larger studies are warranted to investigate underlying mechanisms and subtype-specific alterations.

Neither our study design, nor our data allows conclusions regarding underlying pathophysiological processes. As concentrations of DHEAS numerically declined across the three groups, one might speculate about the role of glucocorticoid excess within the range defined as normal. On the other hand, insulin resistance *per se* might be a driver of tumor growth in adrenal incidentaloma patients (37).

Interestingly, intrahepatic lipid content was not increased in MACS, despite the adverse metabolic profile. The number of patients passing the cut-off (>5.5%) for the diagnosis of hepatic steatosis was also comparable between the three groups. We have recently reported a significant reduction in intrahepatic liver fat following short-term medical treatment with evening doses of metyrapone in patients with MACS, suggesting an immediate impact of altered HPA axis signaling (38). Nevertheless, as obesity is the most relevant risk factor for steatotic liver disease (39), matching for BMI might have attenuated the effects of mild hypercortisolism in inter-group comparisons.

Our study is limited by the relatively small patient number per cohort. However, we applied state-of-the art metabolic imaging methodology to enhance sensitivity and limit inter- and intra-observer variability as observed in previously performed placebo controlled trials (40). Furthermore, the cross-sectional and exploratory study design does not allow to draw conclusions on potential causative effects. The control cohort was selected retrospectively from previously published protocols and a systematic hormonal assessment of adrenal function was not performed. Especially the missing concentrations of ACTH and cortisol post DST are two important limitations. Additionally, the exclusion of participants with a history of cardiovascular disease or the active intake of a medication affecting glucose or lipid metabolism from the control group might impose a selection bias and could impact the observed differences between groups.

To conclude, in our study MACS is associated with an increase in epicardial and paracardial adiposity and an adverse cardio-metabolic risk profile. Since epicardial fat is a well-known mediator for atherosclerosis, microvascular dysfunction and myocardial fibrosis, we assume that it could contribute to the increased cardiovascular mortality in patients with MACS. The higher amount of epicardial fat and the worse metabolic profile in NFA compared to CON warrants further investigation whether these finding are in line with the traditional concept of “non-functioning” adenomas.

## Data Availability

The datasets presented in this article are not readily available because the dataset is not publicly available due to patient privacy. Requests to access the datasets should be directed to Peter Wolf, peter.wolf@meduniwien.ac.at.

## Glossary

¹H-MRS: Proton magnetic resonance spectroscopy
ACTH: Adrenocorticotropic hormone
ALAT: Alanine aminotransferase
ASAT: Aspartate aminotransferase
AUC: Area under the curve
BCAA: Branched-chain amino acids
BMI: Body mass index
BSA: Body surface area
CKD-EPI: Chronic Kidney Disease Epidemiology Collaboration
CO: Cardiac output
CON: controls
CYP3A4: cytochrome P450 3A4
CT: Computed tomography
DHEAS: Dehydroepiandrosterone sulfate
DST: Dexamethasone suppression test
EAT: Epicardial adipose tissue
ECG: Electrocardiogram
ED: End-diastolic
EDV: End-diastolic volume
EF: Ejection fraction
eGFR: Estimated glomerular filtration rate
ES: End-systolic
ESV: End-systolic volume
GLP-1: Glucagon-like peptide 1
HbA1c: Glycated hemoglobin
HDL: High-density lipoprotein
HOMA_IR: Homeostatic Model Assessment for Insulin Resistance
HPA: Hypothalamic, pituitary, adrenal
IHL: Intrahepatic lipid content
LC-MS/MS: Liquid chromatography tandem mass spectrometry
LDL: Low-density lipoprotein
MACS: Mild autonomous cortisol secretion
MRI: Magnetic resonance imaging
NFA: non-functioning adenoma
OGIS: Oral glucose insulin sensitivity index
OGTT: Oral glucose tolerance test
PAT: Paracardial adipose tissue
SGLT2: sodium glucose linked transporter 2
SV: Stroke volume
ULN: Upper limit of normal
VIF: Variance Inflation factor
VLDL: Very-low-density lipoprotein
WC: Waist circumference
W.s.: Water signal.
