# Supplementary material for: Adverse cardio-metabolic risk profile and epicardial adiposity in patients with adrenal adenoma: a cross-sectional study
Source: Front Endocrinol (Lausanne). 2026 Aug 5;17:1898271. doi: 10.3389/fendo.2026.1898271 (PMC13485594; doi:10.3389/fendo.2026.1898271)

Supplemental Material

Adverse cardio-metabolic risk profile and epicardial adiposity in patients with adrenal adenoma: a cross-sectional study

Helena Niziolek[1], Ivica Just[1], Anna Tosin[1], Clemens Baumgartner[1], Oliver Koldyka[1], Konrad Körmöczi[1], Paul Fellinger[1], Hannes Beiglböck[1], Luise Bellach[1], Oliver Domenig[2], Maximilian Zeyda[3], Hansjörg Habisch[4], Tobias Madl[4,5], Anton Luger[1], Greisa Vila[1], Siegfried Trattnig[6], Alexandra Kautzky-Willer[1], Thomas Scherer[1], Michael Leutner[1], Michael Krebs[1], Martin Krssak[1], Peter Wolf[1]

1) Division of Endocrinology and Metabolism, Department of Internal Medicine III, Medical University of Vienna, Vienna, Austria
2) Attoquant diagnostics, Vienna, Austria
3) Division of Pediatric Pulmonology, Allergology and Endocrinology, Department of Pediatrics and Adolescent Medicine, Medical University of Vienna, Vienna, Austria
4) Division of Medicinal Chemistry, Otto Loewi Research Center for Vascular Biology, Immunology and Inflammation, Medical University of Graz, Austria
5) BioTechMed-Graz, Graz, Austria
6) Department of Biomedical Imaging and Image Guided Therapy, Medical University of Vienna, Vienna, Austria

Supplemental table 1: Post-hoc Dunn's tests were performed for variables with a significant Kruskal Wallis test result. Both unadjusted and Holm-adjusted p-values are displayed. Significant p-values are indicated in bold. Abbreviations: AUC, area under the curve; DHEAS, dehydroepiandrosterone sulfate; HOMA_IR, Homeostasis model assessment of insulin resistance

**Post-hoc analysis**	**z-value**	**Unadjusted p-value**	**Holm adjusted p-value**
**DHEAS**			
MACS vs NFA	-2.399	**0.016**	**0.033**
MACS vs CON	-3.436	**<0.001**	**0.002**
NFA vs CON	-1.849	0.065	0.065
**HbA1c**			
MACS vs NFA	0.733	0.464	0.464
MACS vs CON	2.448	**0.014**	**0.043**
NFA vs CON	1.723	0.085	0.170
**HOMA_IR**			
MACS vs NFA	0.766	0.444	0.444
MACS vs CON	3.027	**0.002**	**0.007**
NFA vs CON	2.238	**0.025**	0.050
**Fasting insulin**			
MACS vs NFA	0.721	0.471	0.471
MACS vs CON	2.821	**0.005**	**0.014**
NFA vs CON	2.078	**0.038**	0.075
**Fasting c-peptide**			
MACS vs NFA	1.051	0.293	0.293
MACS vs CON	2.805	**0.005**	**0.015**
NFA vs CON	1.737	0.082	0.165
**AUC glucose**			
MACS vs NFA	0.980	0.327	0.327
MACS vs CON	3.008	**0.003**	**0.008**
NFA vs CON	2.051	**0.040**	0.081
**AUC c-peptide**			
MACS vs NFA	1.545	0.122	0.244
MACS vs CON	2.573	**0.010**	**0.030**
NFA vs CON	1.056	0.291	0.291
**Gamma-GT**			
MACS vs NFA	1.933	0.053	0.107
MACS vs CON	2.540	**0.011**	**0.033**
NFA vs CON	0.607	0.544	0.544
**Triglycerides**			
MACS vs NFA	2.308	**0.021**	**0.042**
MACS vs CON	2.316	**0.021**	0.062
NFA vs CON	0.031	0.975	0.975
**Mean epicardial adipose tissue**			
MACS vs NFA	2.084	**0.037**	0.074
MACS vs CON	3.907	**<0.001**	**<0.001**
NFA vs CON	1.985	**0.047**	**0.047**
**Mean paracardial adipose tissue**			
MACS vs NFA	0.905	0.366	0.366
MACS vs CON	2.601	**0.009**	**0.028**
NFA vs CON	1.774	0.076	0.152

Supplemental table 2: Post- hoc pairwise comparisons of proportions following significant chi-square test

**Comorbidities**	**p-value**
**Hypertension**	
MACS vs NFA	0.723
MACS vs CON	**0.009**
NFA vs CON	**0.045**
**Dyslipidemia**	
MACS vs NFA	0.192
MACS vs CON	**0.011**
NFA vs CON	0.299
**Prediabetes**	
MACS vs NFA	0.376
MACS vs CON	0.107
NFA vs CON	**0.007**

Supplemental table 3: Correlation analysis using Spearman's correlation coefficient for HOMA_IR and epicardial adipose tissue with relevant clinical outcome variables. Significant p-values are indicated in bold. Abbreviations: ACE-S, angiotensin II / angiotensin I; ATCH, Adrenocorticotropic hormone; AUC, area under the curve, DHEAS, dehydroepiandrosterone sulfate ;DST, dexamethasone suppression test; EF, ejection fraction; ES, end-systolic; ESV, end-systolic volume HDL, high density lipoprotein, HOMA_IR, Homeostasis model assessment of insulin resistance ;LDL, low-density lipoprotein; OGIS, Oral Glucose Insulin Sensitivity; PRA-S, angiotensin I + angiotensin II; w.s., water signal

	**HOMA_IR**		**Epicardial adipose tissue**	
Variables	R	**p-value**	R	**p-value**
Age, in years	-0.089	0.454	0.195	0.111
Waist circumference, mm	0.652	**<0.001**	0.451	**<0.001**
Weight, kg	0.451	**<0.001**	0.285	**0.019**
Body fat percentage, %	0.427	**0.002**	0.389	**0.006**
Total body fat mass, kg	0.575	**<0.001**	0.441	**0.001**
Total body fat free mass, kg	0.265	0.058	0.049	0.730
Fasting glucose, mg/dL	0.606	**<0.001**	0.281	**0.020**
HbA1c, %	0.129	0.277	0.167	0.174
HOMA_IR	1	<0.001	0.427	**<0.001**
Fasting insulin, µlU/mL	0.988	**<0.001**	**0.433**	**<0.001**
Total cholesterol, mg/dL	-0.212	0.072	-0.284	**0.019**
HDL cholesterol, mg/dL	-0.345	**0.003**	-0.197	0.107
LDL cholesterol, mg/dL	-0.139	0.242	-0.249	0.041
Triglycerides, mg/dL	0.508	**<0.001**	0.319	**0.008**
Systolic blood pressure, mmHg	0.042	0.730	0.139	0.264
Diastolic blood pressure, mmHg	0.104	0.390	0.27	0.027
OGIS,ml·min^{-1}·m^{-2}	-0.682	**<0.001**	-0.264	0.037
Fasting C-peptide,ng/mL	0.868	**<0.001**	0.431	<0.001
$AUC_{Glucose}$, mg*min/dL	0.182	0.136	0.279	0.028
$AUC_{Insulin}$, µlU*min/mL	0.738	**<0.001**	0.318	0.012
$AUC_{C\text{-}Peptide}$, ng*min/mL	0.666	**<0.001**	0.332	0.008
ACTH, pg/mL	-0.246	0.084	-0.353	0.012
DHEAS, µg/mL	-0.277	0.041	-0.316	0.022
Serum-Cortisol, µg/dL	-0.164	0.222	-0.038	0.784
Hepatic lipid content, % of w.s.	0.538	**<0.001**	**0.255**	**0.036**
Intramyocardial lipid content, % of w.s.	0.14	0.248	0.25	0.043
Total visceral fat mass, ml	0.532	**<0.001**	0.331	0.020
Total subcutaneous fat mass, ml	0.564	**<0.001**	0.431	0.002
EF, %	0.166	0.179	0.125	0.327
EDV, ml/m2	-0.129	0.304	-0.035	0.786
ESV, ml/m2	-0.191	0.124	-0.119	0.354
Stroke, ml/m2	0.024	0.847	0.018	0.887
Cardiac index, L/min/m2	0.047	0.707	-0.021	0.871
Left ventricle myocardial mass average g/m2	0.154	0.219	0.195	0.129
Angiotensin II (1-8), pmol/L	0.328	0.022	0.033	0.822
Angiotensin I (1-10), pmol/L	0.283	0.049	0.098	0.503
Aldosterone, pmol/L	0.386	0.006	-0.093	0.522
Aldosterone-to-Angiotensin II-ratio	-0.153	0.294	-0.212	0.144
PRA-S, pmol/L	0.348	0.015	0.074	0.610
ACE-S	0.087	0.550	-0.018	0.905
Cortisol after DST, µg/dL	0.039	0.789	0.197	0.174

Supplemental table 4: Linear regression models were applied to assess epicardial adipose tissue and HOMA_IR with group status and clinically relevant covariates. Abbreviations: DHEAS, dehydroepiandrosterone sulfate

**Epicardial adipose tissue, mm^2**	Estimate	95%-KI	p-value
NFA	-264,704	-450,761-(-78,647)	**0.006**
CON	-344.2540	-556.228-(-132,279)	**0.0019**
Waist circumference, cm	11.639	5.450-17.827	**<0.001**
Age, years	6.226	-3.425-15.877	0.202

**HOMA_IR**	**Estimate**	**95%-KI**	**p-value**
NFA	-0.223	-0.551-0.104	0.178
CON	-0.443	-0.794-(-0.092)	**0.014**
Waist circumference, cm	0.029	0.018-0.039	**<0.001**
Age, years	-0.008	-0.025-0.009	0.333

Supplemental figure 1: Lipidomics analysis with the first column indicating log2(Fold-change) =log2(Median NFA group/Median MACS group), with negative (blue) values indicating smaller values in the NFA cohort compared to the MACS group. Positive (red) values indicate an increase of median values of the NFA group compared to the MACS cohort. The second column indicates the -log10(of calculated p-value for each variable). Significant p-values were indicated in red, while coloring of non-significant p-values was suppressed for easier readability.

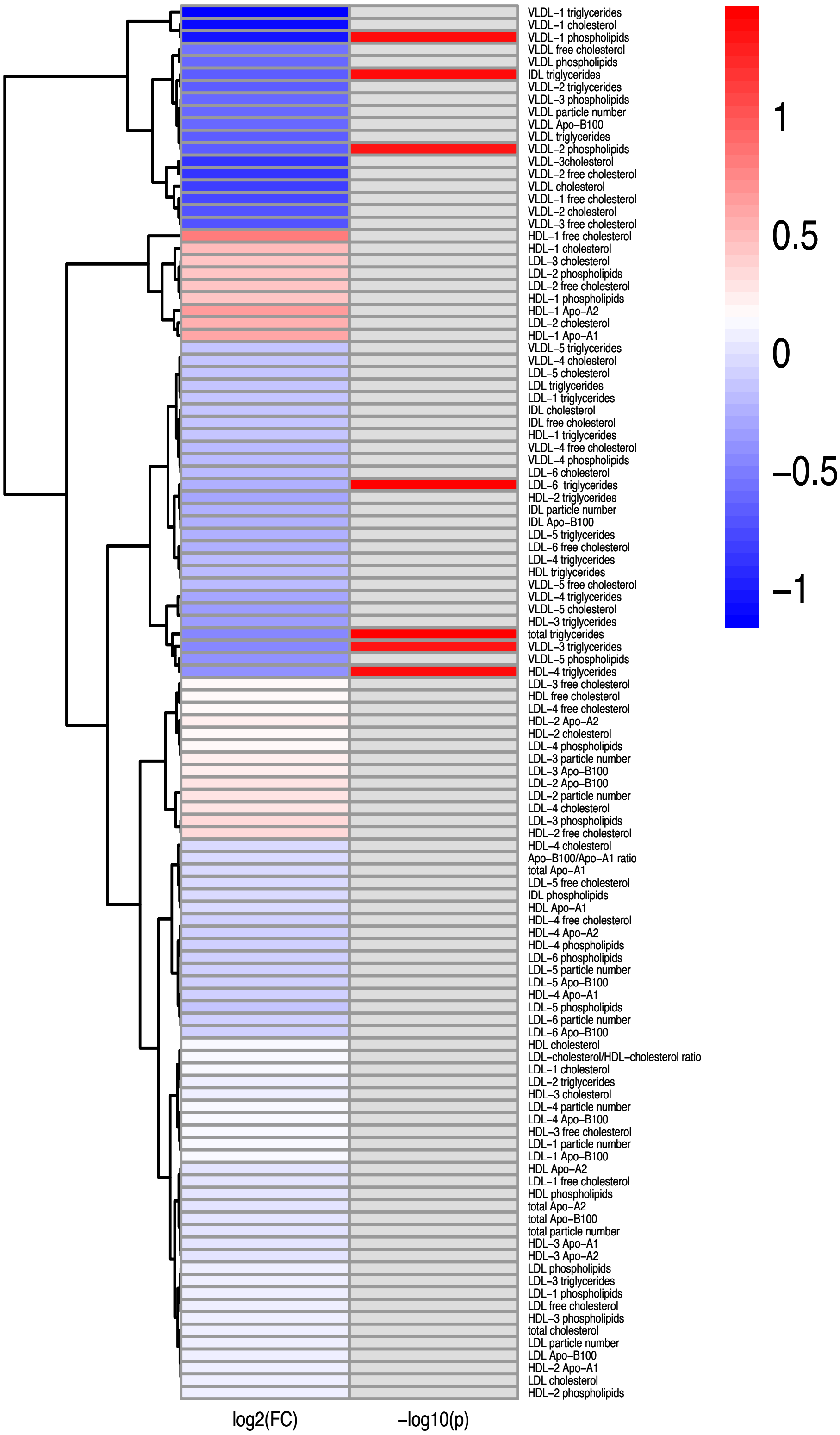

Supplement: Supplementary file 1 [file DataSheet1.pdf]
